# Numerical Modeling of Mechanical Behavior of Functionally Graded Polylactic Acid–Acrylonitrile Benzidine Styrene Produced via Fused Deposition Modeling: Experimental Observations

**DOI:** 10.3390/ma16145177

**Published:** 2023-07-23

**Authors:** Caglar Sevim, Umut Caliskan, Munise Didem Demirbas, Safa Ekrikaya, Mustafa Kemal Apalak

**Affiliations:** 1 Department of Mechanical Engineering, Faculty of Engineering, Niğde Ömer Halisdemir University, Nigde 51240, Turkey; caglar.sevim@ohu.edu.tr; 2 Department of Mechanical Engineering, Erciyes University, Kayseri 38280, Turkey; ucaliskan@erciyes.edu.tr (U.C.); apalakmk@erciyes.edu.tr (M.K.A.); 3 Graduate School of Natural and Applied Sciences, Erciyes University, Kayseri 38280, Turkey; safaekrikaya@gmail.com

**Keywords:** functionally graded material, additive manufacturing, tensile test, finite element model, PLA, ABS, FDM

## Abstract

Functionally graded materials (FGM) have attracted considerable attention in the field of composite materials and rekindled interest in research on composite materials due to their unique mechanical response achieved through material design and optimization. Compared to conventional composites, FGMs offer several advantages and exceptional properties, including improved deformation resistance, improved toughness, lightness properties, and excellent recoverability. This study focused on the production of functionally graded (FG) polymer materials by the additive manufacturing (AM) method. FG structures were produced by the fused deposition modeling (FDM) method using acrylonitrile benzidine styrene (ABS) and polylactic acid (PLA) materials, and tensile tests were performed according to ASTM D638. The effects of different layer thicknesses, volume ratios, and total thicknesses on mechanical behavior were investigated. The tensile standard of materials produced by additive manufacturing introduces geometric differences. Another motivation in this study is to reveal the differences between the results according to the ASTM standard. In addition, tensile tests were carried out by producing single-layer samples at certain volume ratios to create a numerical model with the finite element method to verify the experimental data. As a result of this study, it is presented that the FG structure produced with FDM improves mechanical behavior.

## 1. Introduction

FGMs are defined as advanced composites because they eliminate the delamination and separation problem that is a disadvantage of conventional composites. With the use of FGMs, in-plane and transverse thickness direction stresses can be reduced, residual stress distribution can be controlled, and superior thermal properties without delamination and reduced stress intensity factors can be obtained [1,2,3,4,5]. Therefore, it has been the subject of many studies on aviation and energy for the last 10 years [6,7]. However, effective FGM manufacturing processes are still incomplete, and real applications are rare. There are three methods in the literature regarding production processes. The first one is deposition-based methods used for FGM coatings [8,9,10,11]. The second method is the liquid state method and involves the external addition of reinforcement particles to the melted material [12,13,14,15,16]. The last method is solid-state methods, and these are AM methods with powder metallurgy [17,18,19,20,21,22,23].

Recently, the production of polymer composites and FGMs has been the subject of research and development for reasons such as high strength, easily customizable product properties, flexible manufacturing processes, high resistance to corrosion or erosion, and low cost. In addition, solid-state methods in polymer composite and FGM manufacturing facilitate the fabrication of complex parts and offer low cost and high accuracy. Among AM techniques, the FDM technique is the most widely used in the literature for composite and FGM production [24,25]. Some studies carried out in recent years in which FGM production was carried out with the FDM technique and its mechanical properties were investigated are summarized below.

Su et al. [26] produced ABS and PLA polymers as FG using the FDM. They used X-ray computed tomography to evaluate the air gap distribution, Young’s modulus, strain history variation, and the unfilled fraction of the fabricated material and performed the tensile tests. They showed that the FDM method is suitable for FGM production and emphasized that the printing process of the material should be optimized. Wang et al. [27] proposed a new method using continuous welding filaments to 3D-print carbon-fiber (CF)/glass-fiber (GF) polyetheretherketone (PEEK) materials as FG. They also conducted a series of 3D-printing experiments to validate their design method. They showed that 3D-printed FGMs composed of CF/GF-PEEK composites had good interlayer bonding performance and excellent toughness. They stated that the elongation at break of FGMs prepared in this study increased by 150% compared to fiber-reinforced PEEK composites. Salem et al. [28] produced FG beams with PLA and ABS/nylon materials using the 3D solid-printing technique and investigated their post-buckling behavior. Theoretical and numerical models were developed to validate their results. They performed material topology optimization for the effect of energy released during bending mode transitions in FGM beams on material functions. Subramaniyan et al. [29] fabricated WPC/ceramic-PLA materials as FG with FDM and examined their tensile, compression, bending, impact, and hardness properties. They emphasized that the tensile strength of the FG structure is superior to weaker composites (such as wood polymer composite) and comparable to flexible materials (such as ceramic-reinforced polylactic acid). Anthony Xavior et al. [30] produced ABS material with variable density in the form of FGM with AM technology. They investigated the mechanical strength of FGM material under tension, bending, and compression. They stated that despite the decreasing density, the decrease in strength is minimal and can be used in large tensile and bending loads. They emphasized that it is only used to a certain extent in applications involving large compression loads. Hasanov et al. [31] produced and characterized FGM polymers (PLA-ABS) with FDM. They confirmed that the printing temperature and the volume fraction had an effect on the tensile test results. They used a data-driven approach to construct a linear regression model to formulate input data in FEA. They emphasized that 3D AM provides a low-cost manufacturing process and that this method is a unique method for fabricating FGM structures. Hong et al. [32] proposed a microstructure-dependent magneto-electro-elastic functionally graded porous (MEEFGP) beam model. The model incorporates the extended modified couple stress theory to account for the microstructure effect. Analytical solutions are obtained for the static bending and wave propagation behaviors of the beam model. The results confirm the presence of the microstructure effect and the magneto–electro–elastic multi-field coupling effect. Jędrysiak [33] investigated the behavior of slender elastic nonperiodic beams. They employed the tolerance modeling method to derive the general and standard tolerance models, taking into account the size of the microstructure. The obtained results provide valuable tools for exploring the effect of microstructure size on vibrations. Hasanov et al. [34] produced FGM by AM using carbon fiber and ABS materials and investigated the numerical and experimental characterization of these materials. They performed tensile testing to characterize the interface strength in direct and cascading models and stated that grading increases the strength of ABS material. They presented the results for different compositional gradient values and different interface models.

In light of all these studies, studies are continuing to show the efficiency of the FDM method in the production of FGMs and propose different numerical modeling methods in the light of experimental tests.

In this study, tensile tests were applied to FG structural samples produced by the FDM technique, and the effect of composition gradient on mechanical strength was investigated. A deficiency in AM was observed in the ASTM standards recommended for the relevant tests, and a parametric study was presented in this context. Layer thickness, volume ratio, and total thickness, which are the basic parameters affecting the determined layer structures, were examined. The determined material properties were transferred to the finite element code for material modeling. The tensile behavior of FG structures was also confirmed by numerical analysis.

## 2. Material and Methods

### 2.1. Experimental Process

**Figure 1:**
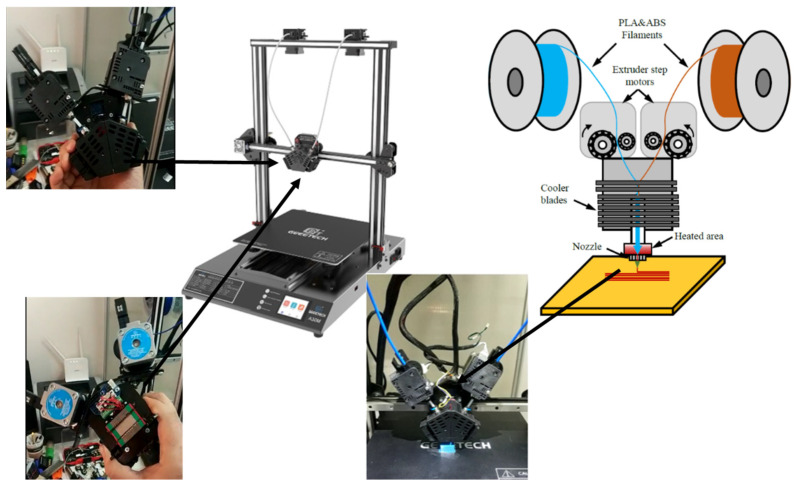
The design of the 3D printer used in this study: 2-in/1-out print head system, electronic system and linear ball carriage, and view of the print head during printing on the printer.

PLA and ABS filament supplied by Filameon were used in this study [35]. A 3D printer with linear sliding ball bearings in each axis of motion was used. The printer has two inlet and one outlet printing nozzles that print by containing different materials and allow for adjustment of the mixing ratios using G-CODEs. The materials entering the nozzle in the desired proportions mix and exit from a single outlet. The printing principle of the 3D printer with two inputs and one output is shown in Figure 1.

The printer has linear plain bearings and ball carriers in the X, Y, and Z axes. This ensures stable and accurate movements of the print head. The filament feed was performed directly by the feeder motor unit on the *X*-axis movable print head. For this purpose, as shown in Figure 1, the print head has been modified to position the extruder motors directly above the hotend. Each test specimen was produced individually and under identical conditions. Details about the printing parameters used for each test specimen are presented in Table 1. 

Since the chemical structures of PLA and ABS materials are different from each other, this necessitates that printing conditions should be designed under different conditions. Therefore, these changing conditions were taken into account when producing a functionally graded composite structure. These processes were neglected in most of the studies. For the best printing properties, the printing nozzle temperature and table temperature were gradually increased as the ABS content in the composite structure increased, and the cooling fan was turned off when the ABS content exceeded 20%. The designed test specimens were converted to .stl format via the CAD program, and GCODEs were created with Ultimaker CURA V4.10.0 slicing program [36]. Mixture ratios and temperature adjustments were adjusted by editing the GCODEs by ourselves. The properties of PLA and ABS materials produced under the conditions described above are detailed in Table 2. This study investigated the tensile strengths of the specimens produced by determining the functionally specific volume ratios. Since the ideal printing temperatures of PLA and ABS are slightly different from each other, when printing with 100% PLA, the appropriate printing temperature of 205 °C is used, and for a 10% ABS ratio, the nozzle temperature is set to 215 °C, while for ratios above 10%, the nozzle temperature is adjusted to 218 °C. When printing with 100% ABS, a nozzle temperature of 220 °C is used. Although Table 2 indicates an ideal bed temperature of 60 °C for PLA, in the FGM structure where ABS is present, a bed temperature of 80 °C is used for all prints.

**Figure 2:**
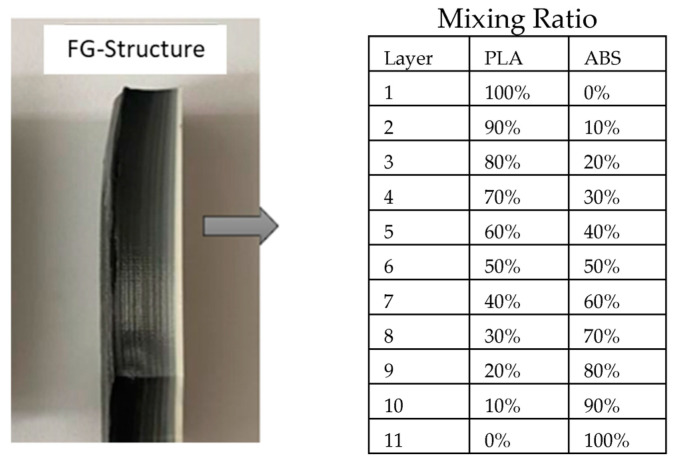
Mixing ratios for the functional gradient used in preliminary studies.

**Figure 3:**
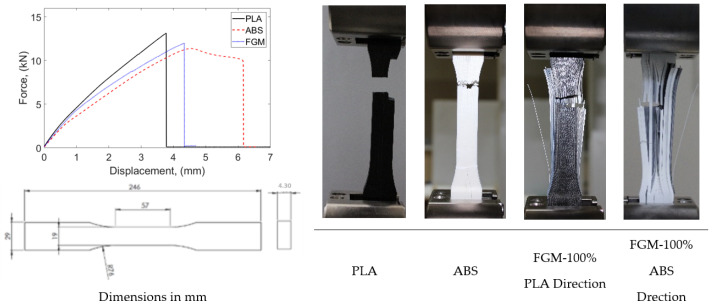
Tensile test results of 11-layer FG structure, PLA, and ABS specimens used in preliminary studies.

**Figure 4:**
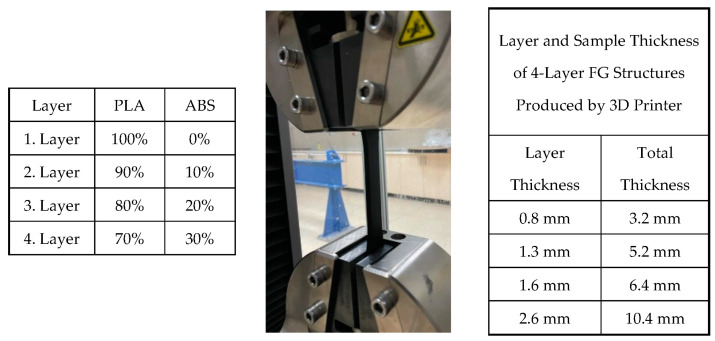
FG structures samples, which were subjected to tensile tests according to ASTM D638 standard.

The variation in the compositional gradient exponent was considered linear in the grading of ABS and PLA materials. FG samples for the 11 layers given in Figure 2 were produced in all ratios. However, delamination occurred in compositions with less than 70% PLA, as seen in Figure 3. Therefore, in the sample production performed according to the ASTM D638 standard [37], the volume ratios in the FG production were determined as given in Figure 4.

**Figure 5:**
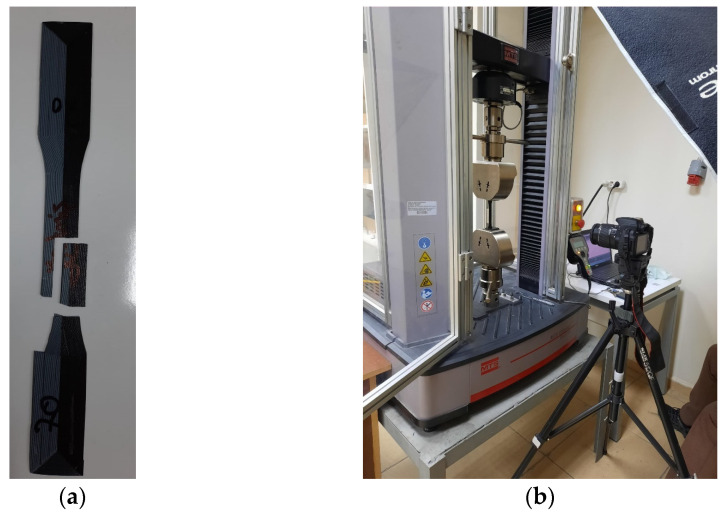
Experimental system. (**a**) Tensile test specimen. (**b**) Test equipment.

Considering the ASTM D638 standard, all dimensions change with changing thicknesses. For example, the changes of other geometric dimensions according to the change in thickness are described as Types I, II, III, IV, and V. Types I and II are defined for thicknesses of 7 mm or less, Type III for thicknesses between 7 and 14 mm, and Types IV and V are defined for materials with a thickness of 4 mm or less with a different geometric shape. Since the materials produced do not exhibit isotropic behavior, different parameters were planned and produced. In Figure 5, layer thicknesses are given as 0.8, 1.3, 1.6, and 2.6 mm according to the planned functional gradient. The total specimen thickness resulting from these layer thicknesses represents two different types of ASTM standards. Accordingly, specimens were produced for both the same type of specimens and different thicknesses and subjected to tensile tests.

As a result of the specified layer thicknesses, the total thicknesses of the samples were 3.2, 5.2, 6.4, and 10.4 mm. The reasons for the selection of these thicknesses are as follows: specimen thicknesses vary as a result of the layer thickness effect in FG structures whose tensile behavior was tried to be determined. According to the layer and total thicknesses to be determined, the specimen dimensions according to ASTM standard will emerge. In this respect, considering the printing parameters, the layer wall thickness is 0.4 mm. It was considered that single-walled productions may cause problems in terms of performance and a structure with two wall thicknesses was preferred as the first layer thickness and decided as 0.8 mm. After this stage, a layer thickness of two times 1.6 mm was first preferred to investigate the effect of layer thickness. At this stage, the sample thickness increased from 3.2 mm to 6.4 mm. This size range corresponds to Type I of the relevant ASTM standard. With the increase in layer thickness, the size range in the ASTM standard moves to Type III. In order to determine the differences between these dimensions, Type I was dimensioned with a layer thickness of 1.3 mm (total thickness 5.2 mm) and Type III with a layer thickness of 2.6 mm (total thickness 10.4 mm). In this way, the thickness increase within the same Type, i.e., within the same dimensions, will be analyzed, and the thickness increase between different Types will be analyzed.

**Figure 6:**
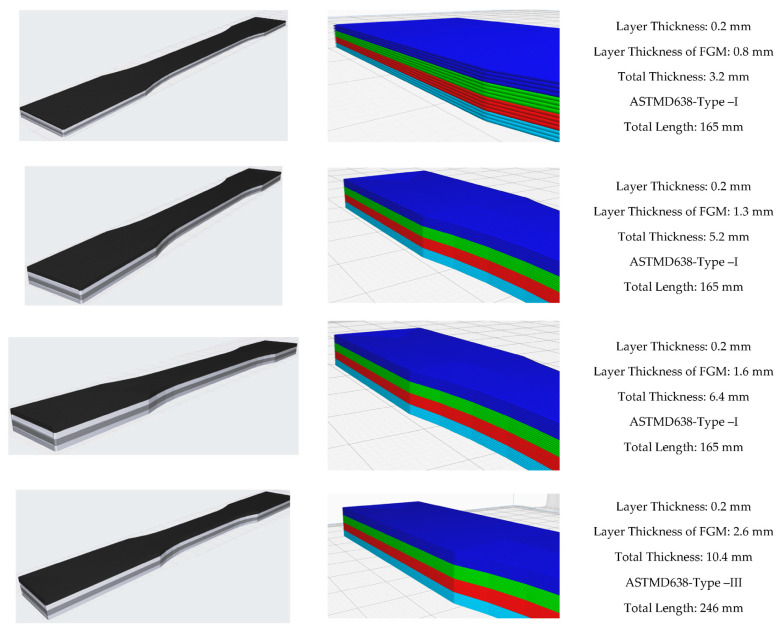
Geometric properties and layer configuration of the functionally designed structures.

To determine the sizing in Figure 4, an 11-layer FG structure was fabricated (Figure 2) and subjected to tensile testing, as shown in Figure 3. According to this test, delamination was observed in the FG structure. The layers did not adhere to each other. For this reason, it was aimed to prevent this situation by reducing the number of layered layers. As can be seen from the figure after the tensile test, the reduction of ABS layers significantly prevented delamination’s. Figure 5 shows the experimental test setup and the fracture geometry of the FG structure subjected to tensile test. Tensile tests were performed under a tensile speed of 1 mm/min. The tensile test equipment is MTS brand and includes clamping jaws, and the load capacity of the device is 50 kN. The tests were recorded with a camera and the related deformation images were analyzed. Figure 6 shows the printed images, layer changes and residuals of the designed structures. 

### 2.2. Numerical Modeling

The tensile behavior of FG structures was determined experimentally with all affecting parameters. The results that emerged in line with ASTM standards were in the direction that all parameters affect tensile behavior. The experimental results were transferred to a finite-element code and the FG structure was modeled. Finite-element analyses were performed using Abaqus/Standard the version number of 6.14 [38].

The layer structure was modeled by transferring the solid models of the tensile specimens to the finite elements. All samples were designed as eight layers with different layer thicknesses. The mesh structure was created with the C3D8R solid element. Each layer was transferred to the finite-element code as a result of the tensile tests carried out in its own thickness. The experimental tensile test concept was transferred to the numerical model similarly. The total stress is defined from the total elastic stress as follows [38]:

(1) $$\sigma =D^{el}\varepsilon^{el}$$

where σ is the total stress (the “real” or Cauchy stress in finite strain problems), *D^el^* is the fourth order elasticity tensor, and *ε^el^* is the total elastic strain. The simplest form of linear elasticity is the isotropic case and the stress–strain relationship is given by [38]:

(2) $$\left\{\begin{array}{c} \varepsilon_{11}\\ \varepsilon_{22}\\ \varepsilon_{33}\\ \gamma_{12}\\ \gamma_{13}\\ \gamma_{23} \end{array}\right\}=\left[\begin{array}{cccccc} 1/E & -v/E & -v/E & 0 & 0 & 0\\ -v/E & 1/E & -v/E & 0 & 0 & 0\\ -v/E & -v/E & 1/E & 0 & 0 & 0\\ 0 & 0 & 0 & 1/G & 0 & 0\\ 0 & 0 & 0 & 0 & 1/G & 0\\ 0 & 0 & 0 & 0 & 0 & 1/G \end{array}\right]\left\{\begin{array}{c} \sigma_{11}\\ \sigma_{22}\\ \sigma_{33}\\ \sigma_{12}\\ \sigma_{13}\\ \sigma_{23} \end{array}\right\}$$

This material model is very widely used as a rate-dependent or rate-independent model in plasticity calculations and has a particularly simple form. Due to this simplicity, the algebraic equations associated with the integration of the model can be easily developed in terms of a single variable, and the material stiffness matrix can be written explicitly. This results in a particularly efficient code. For ease of implementation, it is assumed that all quantities not explicitly associated with a time point are evaluated at the end of the increment. The von Mises yield function associated with the flow means that there is no volumetric plastic strain; since the elastic bulk modulus is quite large, the volume change will be small. Thus, we can define the volumetric strain as

(3) $$\varepsilon_{vol}=trace\left(\varepsilon \right)$$

and, hence, the deviatoric strain is

(4) $$e=\varepsilon -\frac{1}{3}\varepsilon_{vol}I\;$$

Using the standard definition of corotational measures, this can be written in integrated form as

(5) $$\varepsilon =\varepsilon^{el}+\varepsilon^{pl}$$

The elasticity is linear and isotropic and, therefore, can be written in terms of two temperature-dependent material parameters. For the purpose of this development, it is most appropriate to choose these parameters as the bulk modulus, *K*, and the shear modulus, *G*. These are computed readily from the user’s input of Young’s modulus, *E*, and Poisson’s ratio, ϑ, as

(6) $$K=\frac{E}{3\left(1-2\vartheta \right)}$$

and

(7) $$G=\frac{E}{2\left(1+\vartheta \right)}$$

**Figure 7:**
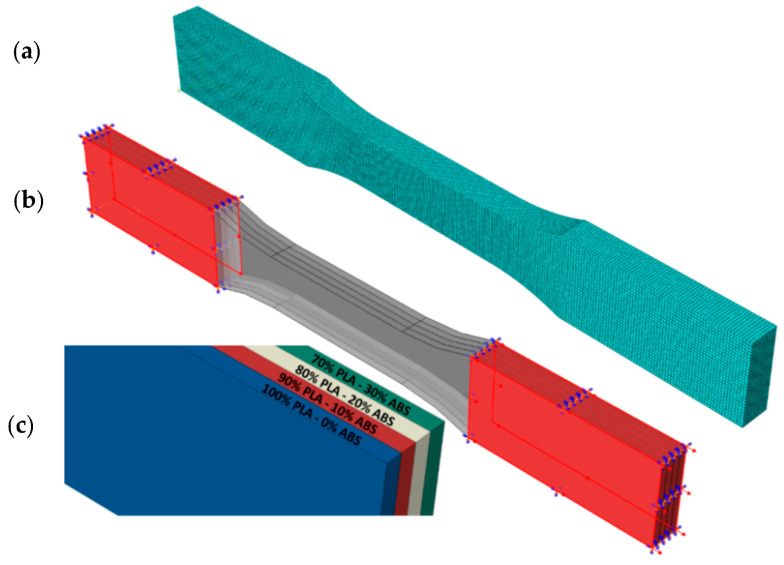
Finite element model of FG specimen: (**a**) mesh model, (**b**) boundary conditions, (**c**) layer mix ratios.

**Figure 8:**
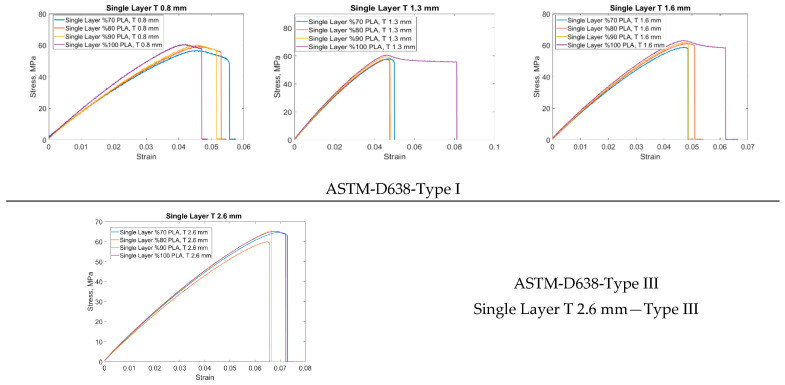
Tensile stress–strain curves of single layers of FG structure manufactured according to ASTM D638 Type I and III.

The outer surfaces of one end of the sample were fixed in all directions, and the other end was displaced according to the experimental displacements. The reaction forces were calculated by reading from the driven nodes. The detailed finite element model is given in Figure 7. Analyses were performed for four different FG configurations. The material model was elastoplastic, and no damage description was considered. The test results for the single layer given in Figure 8 were used.

Figure 8 shows the tensile test results of single-layer specimens with different functional gradients produced according to ASTM D638 Type I and III. The single-layer specimens have thicknesses of 0.8, 1.3, 1.6, and 2.6 mm. The functional gradient ratios are 70% PLA/30% ABS, 80% PLA/20% ABS, 90% PLA/10% ABS, and 100% PLA/0% ABS. The thicknesses of the sample produced for Type I are 0.8, 1.3, and 1.6 mm, while the lengths are 165 mm. The thickness of the sample produced for Type III is 2.6 mm while the length is 246 mm. Compared to Type I, the strain of the 0.8 mm thick specimen increased at similar stress levels with ABS reinforcement. In the 1.3 mm thick specimen, approximately 0.08 strain was measured in the 100% PLA specimen, while it decreased to around 0.05 with ABS reinforcement. Similar results were obtained for the 1.6 mm thick specimen. Compared to Type III, the 100% PLA sample with a layer thickness of 2.6 mm gave higher results in terms of both strain and stress.

## 3. Results

In this study, a parametric study was carried out to develop a numerical model of the FG structure produced by the FDM technique and to model its tensile behavior. The tensile test behavior of the produced specimens and the effect of gradation on mechanical strength were investigated. The main parameters affecting the tensile test results such as layer thickness, volume fraction, and total thickness were also investigated. The main reason for the parametric study in this paper is that the dimensional variations in the ASTM standard for rigid plastics are not suitable for AM. Specimens were designed according to the dimensional parameters in the standard, and each variable parameter was transferred to the numerical model. The creation of a meaningful numerical model will avoid the uncertainties in the ASTM standard and will shed light on many parameters to be studied. The dimensional parameters in the ASTM D638 standard vary primarily according to the total thickness. The total thickness variation is determined by the AM method and the layer thickness decision of the FG structure to be formed. With the development of technology, many filaments containing different materials suitable for AM are available today. With the FG structure proposed in this study, it is possible to produce new material behaviors with many desired properties. 

**Figure 9:**
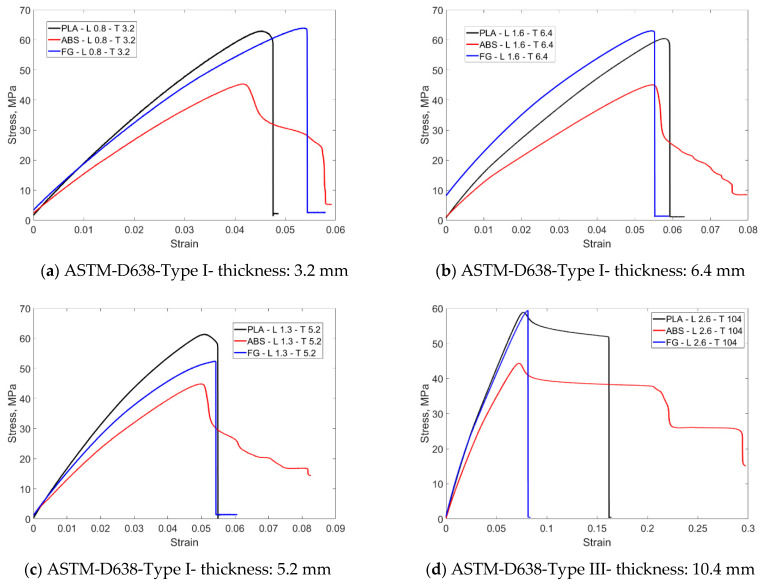
Comparison of tensile stress–strain curves of FG structure, with ABS and PLA specimens manufactured according to ASTM D638 Type I and III.

The stress–strain behavior of FG structures against PLA and ABS as a result of tensile testing according to two different ASTM standards is given in Figure 9. Figure 9a shows the stress–strain behavior of FG, PLA and ABS materials for Type I for layer thickness 0.8 mm and specimen thickness 3.2 mm. The expected feature is that the FG structure exhibits a property between the other two materials. However, at these dimensions, higher strain was obtained at a similar stress level with the FG structure. A successful FG structure configuration has been demonstrated. When the thickness was doubled (Figure 9b, thickness 6.4 mm), the FG structure exhibited similar strain behavior to the other materials. However, an increase in the stress level is observed. These two thicknesses were made for ASTM D638 Type I for layer thickness and material variation. Considering the other thicknesses, a comparison was made between Types I and III. Specimens were manufactured and tensile tested for layer thicknesses of 1.3 and 2.6 mm and total thicknesses of 5.2 and 10.4 mm (Figure 9c,d). In the specimen with a layer thickness of 5.2 mm, the FG structure showed tensile behavior exactly in the middle of PLA and ABS at the same strain. When Type III was switched to Type III, the elongations were higher with the increase in the specimen length. With the FG structure, a more rigid material was obtained at the PLA stress level. The rupture occurred at the highest stress level. In general, ABS stress levels were around 40 MPA, and PLA stress levels were around 60 MPA.

**Figure 10:**
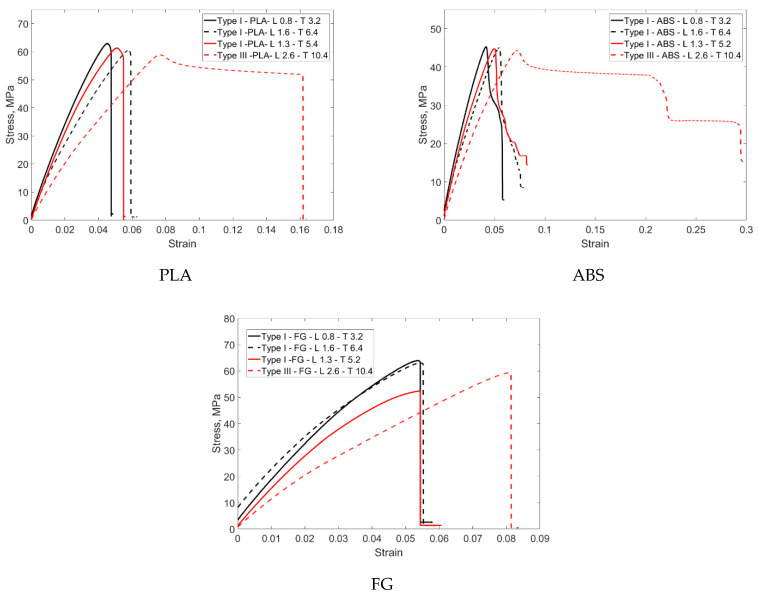
Effect of ASTM D638 Type I and Type III on tensile stress strain behavior for PLA, ABS, and FG structures.

Figure 10 shows the stress–strain graphs obtained as a result of thickness variations for PLA, ABS, and FG specimens. The graphs were organized as thickness comparison for Type I and thickness comparison for Type I and III. When the tensile test results of the samples produced from PLA-based material are compared, it is seen that they exhibit similar stress levels of around 60 MPa. In this context, it can be argued that this material shows a behavior close to isotropic behavior. However, when the test was analyzed for Type III, it was seen that the strain increased by 1% from 0.06% to 0.16%. In this context, the increase in material thickness has a serious effect on the results. This result is evident with all thickness increases, albeit partially. When the tensile test results for ABS material are analyzed according to the thickness increase, the stress levels are around 45 MPa. Similar strain behavior is observed for Type I, whereas strain increases are from 0.05 to 0.3 for Type III. In this context, the thickness increase in this material, which shows more elongation than PLA, affects the results more significantly. This situation is also related to the ductility of the material. This problem was eliminated in the FG structure and almost isotropic material behavior was obtained, especially when compared to Type I. Stress–strain data were obtained at 3.2 and 6.4 mm thicknesses. When Type I and Type III are compared, there is a significant increase in strain with increasing thickness and a partial increase in stress. However, there was no elongation at the break in the materials.

**Figure 11:**
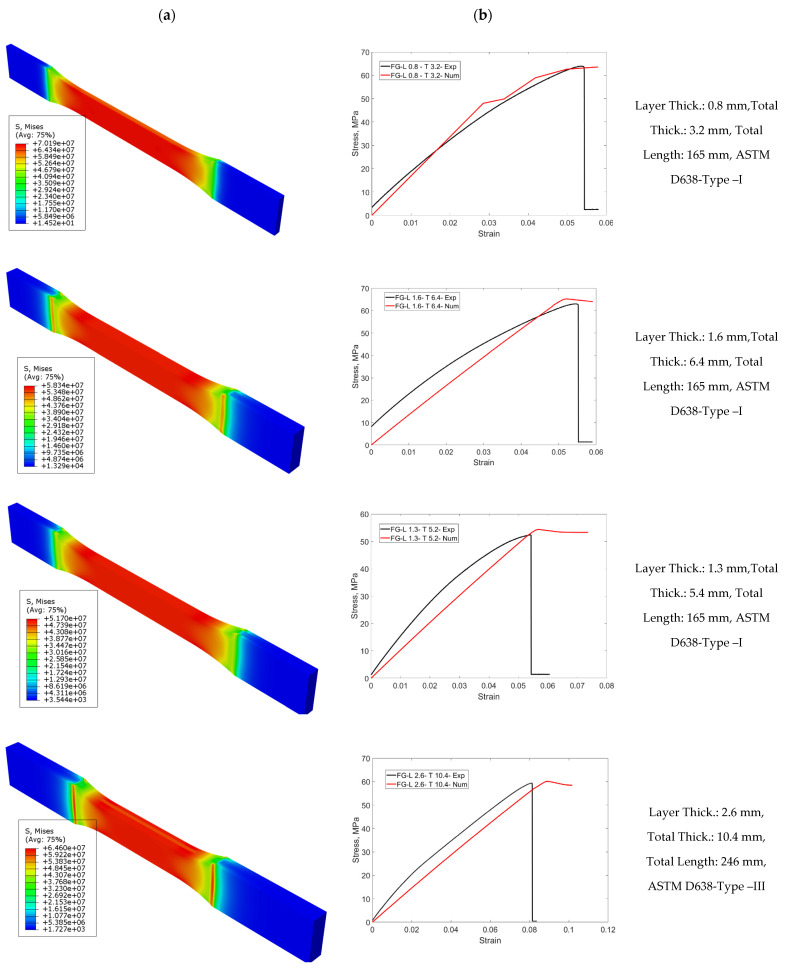
Comparison of numerical and experimental tensile stress–strain behavior of FG structure for ASTM D638 Type I and III standards: (**a**) stress distribution and (**b**) stress-strain curves.

In Figure 11, numerical tensile test results of FG specimens are given as a von Mises stress distribution. Numerical stress-strain results are compared with experimental results. Four different FG configurations were selected according to ASTM standards and different types were compared. The usability of the obtained experimental results as a numerical material model is demonstrated. After obtaining single-layer results, it is proved that finite-element analysis is a serious alternative for designs, saving time and cost. The first configuration has a layer thickness of 0.8 mm and a total thickness of 3.2 mm. The average approximate modulus of elasticity of the structure was calculated to be 1.5 GPa. The stress distribution on the structure was in the same direction as the stress–strain diagram. Experimental and numerical tensile test graphs have similar behavior. The second FG configuration has a total thickness of 6.4 mm and a layer thickness of 1.6 mm. The average modulus of elasticity was measured to be 1.43 GPa. The curve trend associated with the experimental tensile behavior is similar. For Type I, a similar modulus of elasticity was obtained with increasing thickness. The other FG configuration has a total thickness of 5.2 mm and a layer thickness of 1.3 mm. The average and approximate modulus of elasticity was calculated as 1.43 GPa. 

The configuration for Type III has a layer thickness of 2.6 mm and a total thickness of 20.8 mm. The modulus of elasticity was calculated to be 1.1 GPa. The difference in these results is a result of AM. The non-isotropic structure and the production parameters vary according to the dimensions. In this study, this uncertainty in AM has been significantly emphasized. As the thickness increased, the material became resistant to rupture and elongation increased. However, there was no increase in the stress level, resulting in a decrease in the elastic modulus. ABS makes the structure more ductile. In this respect, it is seen that the stress level is higher in PLA-rich final layers.

## 4. Discussion

In this study, each layer forming the FG structure is composed of printing layers with a thickness of 0.2 mm. In future studies, different printing layer thicknesses of 0.4 or 0.8 mm can be selected according to the requirements to examine their influence on mechanical properties. As a result, both the printing time will be reduced and the number of layers and infill fibers that need to adhere to each other will decrease. However, as a disadvantage, the increased diameter of the extruded infill fiber will negatively affect the surface quality. Additionally, in this study, the volumes in the FG structure exhibit a 10% variation. Subsequent studies can consider more precise transitions. Another parameter that affects the mechanical properties of materials printed on 3D printers is the printing speed. High printing speeds result in oscillations during sudden turns, which affect the adhesion of the filament extruded from the nozzle. Further studies can be conducted to determine the optimal printing speed for different volume ratios in PLA-ABS FGM structures. As the printing speed increases, the required printing time will decrease.

## 5. Conclusions

In this study, the tensile behavior of PLA-ABS functional graded structures produced by AM using ASTM D638 was investigated, and a numerical model was proposed for this complex structure. Different manufacturing parameters, such as layer thickness and specimen thickness, were investigated. Parameters suitable for functional grading were determined by a preliminary study. The obtained single-layer tensile results were transferred to the finite-element code and the tensile behavior was modeled numerically. In the combination ratios, it was seen that there was no material combination in the use of more than 30% ABS, and the combination ratios were used as 100% PLA, 90% PLA/10% ABS, 80% PLA/20% ABS, and 70% PLA/30% ABS. Production, tests, and analyses were carried out according to the different dimensions stipulated by the ASTM standard. This is due to the lack of isotropic behavior in AM products. This result is proved by all parameters. It was observed that the elastic modulus varies with each different parameter. The ductility of the material increased with the increase in specimen thickness. The strength of the material improved with the FG structure. In this study, which proposes a new material configuration by reducing the negative aspects of the most widely used AM products, such as PLA and ABS, it is concluded that the ASTM standard is inadequate for AM products and tests should be performed in all changing parameters.

## Figures and Tables

**Table 1:** Printing parameters.

Layer thickness	0.2 mm
Filler fiber thickness	0.4 mm
Wall thickness	0.4 mm
Print speed	30 mm/s
Filling pattern	line (0°)

**Table 2:** Thermal and mechanical properties of ABS and PLA [35].

Properties	PLA	ABS
Filament diameter (mm)	1.75	1.75
Density (gr/cm^3^)	1.24	1.04
Bed temperature (°C)	60	80–100
Nozzle temperature (°C)	205	220
Melt Flow Index (210 °C/2.16 kg)	6	80–120
Tensile strength (MPa)	53	45
Elongation (%)	6	10
Bending strength (MPa)	83	73
Rackwell hardness	108	108
Max service temperature (°C)	55	85

## Data Availability

Not applicable.
